# Functional Properties of Chitosan Oligomers Obtained by Enzymatic Hydrolysis

**DOI:** 10.3390/polym15183801

**Published:** 2023-09-18

**Authors:** Dominika Kulig, Żaneta Król-Kilińska, Łukasz Bobak, Barbara Żarowska, Andrzej Jarmoluk, Anna Zimoch-Korzycka

**Affiliations:** 1 Department of Functional Food Development, Faculty of Food Science and Biotechnology, Wroclaw University of Environmental and Life Sciences, 37 Chelmonskiego St., 51-630 Wroclaw, Poland; zaneta.krol@upwr.edu.pl (Ż.K.-K.); lukasz.bobak@upwr.edu.pl (Ł.B.); andrzej.jarmoluk@upwr.edu.pl (A.J.); anna.zimoch-korzycka@upwr.edu.pl (A.Z.-K.); 2 Department of Biotechnology and Food Microbiology, The Faculty of Biotechnology and Food Science, Wroclaw University of Environmental and Life Sciences, 37 Chelmonskiego St., 51-630 Wroclaw, Poland; barbara.zarowska@upwr.edu.pl

**Keywords:** chitosan, chitooligomers, chitosanase, cellulase, hydrolysis, antimicrobial properties, antioxidant properties

## Abstract

The aims of this study were to obtain chitooligosaccharides (COS) from chitosan (CH) with improved functional properties and comparison of the use of two different enzymes: commercial cellulase (CL) and the dedicated enzyme chitosanase (CS). After enzymatic reaction, chitosan oligomers (NFs) were isolated by methanol into two fractions: precipitate (HMF) and supernatant (LMF). The occurrence of a hydrolysis reaction was confirmed by an increased reducing sugar content and viscosity reduction of chitosan oligomers. CPMAS ^13^C NMR analysis confirmed the dissimilar cleavage mechanism of the enzymes used. LMF and NF fractions were characterised by improved solubility in water (94.56%) compared to the HMF and CH samples (70.64%). Thermogravimetric analysis (TGA) showed that the HMF decomposed in two-stage process while CH, NF, and LMF decomposed in a three-stage process. The greatest mass loss of LMF samples (58.35%) suggests their sensitivity to high-temperature treatments. COS were a mixture of DP (degrees of polymerisation) from 3 to 18 hetero-chitooligomers, with an average Mw of <3 kDa. CL consisted of more low-DP products (DP 3–7) than COS made with CS. LMF characterised by DP~2 showed lower DPPH radical scavenging activity than HMF and NF with DP 3–7. The ability to reduce *Escherichia coli* increased in the given order: LMF > NF > HMF > CH.

## 1. Introduction

The functional characteristics of chitosan depend on its type, molecular weight, and degree of deacetylation. Polysaccharide and its derivatives have been used extensively in the medical, pharmaceutical, cosmetic, food, environmental protection, and biomaterial industries. Nevertheless, chitosan application is hindered by its large molecular weight and presence of rigid crystalline domains, formed by intra-and/or intermolecular hydrogen bonding. These properties are considered responsible for the poor solubility of chitosan in neutral/alkaline pH solutions [1]. The functional and biological properties of chitosan can be improved by irradiating [2], UV light, or partial hydrolysis [3], using different organic solvents [4] or chemical modification [5]. Physical depolymerisation of chitosan occurs under the influence of sound waves (sonication), radiation, or high temperatures. Chemical hydrolysis occurs under the action of inorganic acids (hydrochloric acid, nitric acid) or free radicals (hydrogen peroxide). Enzymatic hydrolysis seems to be the simplest and most convenient way of producing chitooligosaccharides (COS). Because of its low environmental cost, it is considered to be a green technology and a non-hazardous process; the quantity of enzymes used in the hydrolysis process is smaller than that of acids or hydrogen peroxides in chemical hydrolysis. Moreover, it does not require costly equipment and, due to the relatively low reaction temperature, the energy costs of the process are lower than physical methods [6,7,8]. Enzymes catalyse the hydrolytic fragmentation of the chitosan chain. The enzymes most commonly used for this purpose are chitinase or chitosanase, synthesised by bacteria and fungi. Based on their amino acid sequences, chitosanases are classified into seven families of glycoside hydrolases (GH-3, GH-5, GH-7, GH-8, GH-46, GH-75, and GH-80) and further grouped into four classes based on their specificity of cleavage [9]. GH-46, GH-75, and GH-80 contain only chitosanases, while the families GH-5, GH-7 and GH-8 contain other glycoside hydrolases, such as cellulase. Due to the high prices of specific enzymes, their use on an industrial scale is significantly restricted. Other hydrolytic enzymes (lysozyme, papain, cellulase, hemicellulase, pectinase) also have the ability to catalyse the hydrolysis of glycosidic linkages presented in chitosan and can potentially be used in industry [10,11]. The use of a commercial enzyme, cellulase, to obtain chitooligomers, is industrially more economical than chitosanase, so both enzymes were used for comparison in this work. Chitooligosaccharides are of particular interest because of their biodegradability and documented biological and physiological activity: probiotic, antimicrobial, antioxidant, antitumor, and antidiabetic. They also have the ability to stimulate the immune system and accelerate calcium absorption. These features make chitooligosaccharides of interest in both the pharmaceutical and food industries [11,12]. Oxidation processes and microbial growth are the main causes of food spoilage [13,14]. Due to the unique properties of COS, which reduce the growth of bacteria, yeast, and mould, as well as their antioxidant characteristics, they can be used to replace or reduce the use of artificial preservatives and antioxidants currently used in food production and thus affect its quality, safety and shelf life. Examples of possible applications of chitooligomers in foodstuffs are protective coatings and films, microcapsules, or direct applications as food additives. The same biological properties can be used in the pharmaceutical industry, for example, to produce hydrogel wound dressings.

The objectives of this study were to obtain chitooligomers using specific enzymes, chitosanase and the commercially available enzyme, cellulase, as well as compare the products of the fractionated and unfractionated hydrolysis reaction (NF—non-fractioned, LMF—low-molecular-weight fraction, HMF—high-molecular-weight fraction) and their functional properties.

## 2. Materials and Methods

### 2.1. Materials

Chitosan of low molecular weight (DD = 75–85%; Mw = 150 × 10^−3^) and chitosanase (EC 3.2.1.132) from *Streptomyces griseus* with 50 U/mg activity and lactic acid (85% syrup) were obtained from Sigma-Aldrich, Poznan, Poland. Cellulase (E.C. 3.2.1.4) from *Trichoderma reesei* with 127.5 U/mg activity was purchased from Dyadic, Jupiter, FL, USA. Cellulase showed side activities typical of beta-glucanase (33 U/mg).

### 2.2. Enzymatic Hydrolysis of Chitosan and Reaction Progress Control

The chitosan (CH) stock solution was prepared at a concentration of 4% (*w*/*v*) in a 2% lactic acid aqueous solution by stirring at 400 rpm for 20 h. The pH was adjusted to 5.5 by adding an aqueous sodium hydroxide solution (1 M), which caused the dilution of CH to reach the final 3%. Enzymatic solutions: cellulase (CL) and chitosanase (CT) were added in an amount of 1000 U. The reaction with CL was carried out at 55 °C in 4 h, while the reaction with CT was carried out at 37 °C within 4 h. The enzyme kinetic reactions were defined as the amount of enzyme required to produce 1 mM glucose per hour using the DNS method described below.

The reaction progress was determined by measuring the reducing sugar ends using 30% solution of 3,5-dinitrosalicylic acid (DNS) as the reagent and calculated as D-glucose according to Miller et al. [15]. The absorbance of the clear solution was measured at 523 nm using a UV 2601 RAYLEIGH spectrometer (Beifen-Ruili Analytical Instrument, Beijing, China). Hydrolysis progress was also observed as viscosity decreased in function of time. The viscosity measurement was performed on a Haake RheoStress 6000 rotational viscometer (Haake, Karlsruhe, Germany). Measurement was carried out at an optimal constant temperature for each enzyme (37 °C for chitosanase, 55 °C for cellulose and untreated chitosan sample) using a system of coaxial cylinders with conical rotor Z20 DIN. The reaction temperatures were determined on the basis of the data provided by the manufacturer of enzymes preparations, and the temperature profile of each enzyme was checked. The sample of the reaction mixture (8.2 mL) was applied to the cylinder after adding the enzyme to the chitosan solution and was simultaneously analysed on a rheometer throughout the entire hydrolysis process (4 h).

### 2.3. Fractionation

Enzymatic treatment was stopped after 4 h. The CH-hydrolysed solutions were boiled for 30 min to inactivate the enzymes and filtered through G-4 pore size > 10 µm filter membrane under a reduced pressure of 25 mbar to remove it. The purified CH reaction mixture was then concentrated on a vacuum evaporator to obtain 1/5 volume. One part of the obtained solution was freeze-dried to obtain not fractionated hydrolysates mixture (NF) and the second part was used for further modification–fractionation. Precipitation was performed using methanol at a concentration of 80% according to Xie et al. [16]. The CH-methanol solutions were centrifuged to separate the precipitate and supernatant. The precipitate–insoluble, high-molecular-weight fraction (HMF) was washed with 80% methanol two times and freeze dried. The supernatants and washing aqueous methanol solutions were combined, concentrated under vacuum, and finally lyophilised to obtain a soluble, low-molecular-weight fraction (LMF). Unmodified chitosan sample dissolved in 2% lactic acid (CH), NF, HMF, and LMF were poured into polypropylene beakers and frozen at −80 °C for 24 h. Subsequently, the mixtures were lyophilised at vacuum 0.060 mBar/−48 °C for 2 days and stored in a desiccator until analysis.

### 2.4. Apparent Viscosity

The apparent viscosity of the prepared COS sample and the unmodified chitosan sample was investigated by dissolution of freeze-dried samples in water (1% *w*/*v*). Measurement was carried out at a constant temperature (20 °C) using a cone sensor (C60/1° Ti L, diameter 20) and a measuring plate (TMP60 Steel 18/8) in linear distribution CR mode of the Haake RheoStress 6000 rotational viscometer (Haake, Karlsruhe, Germany). The tested samples were conditioned for 3 min at 20 °C. The apparent viscosity was determined as the average value of the measurements made at a constant shear rate of 1.0 s^−1^ during 300 s of analysis.

### 2.5. Water Solubility

Solubility (*S*) was calculated as the percentage of hydrolysates dry matter dissolved during immersion in distilled water for 24 h. For the measurement of solubility, 1 g of each hydrolysate was weighed and dried for 24 h in an air-circulating oven at 60 °C. The dried samples were placed in beakers containing 100 mL of distilled water. The beakers were covered with Parafilm M^®^ and stored at 25 °C for 24 h under constant shaking at 100 mot/min. After 24 h, residual material was filtered by a Munktell filter disc (390 grade) and dried in an air circulating oven (60 °C) for 24 h. The weight of the dissolved dry matter was calculated by subtracting the weight of insoluble solid matter (*Sm*_2_) from the initial weight of the solid matter (*Sm*_1_).

(1) $$S=\frac{{Sm}_{1}-{Sm}_{2}}{{Sm}_{1}}\cdot 100\%$$

### 2.6. pH Measurements

The pH of hydrolysates and unmodified chitosan was measured using a pH/mV/ISE Meter (Seven Multi™ model S40 Mettler Toledo, Columbus, OH, USA) equipped with InLab Routine Pro electrode (Mettler Toledo, Columbus, OH, USA).

### 2.7. Thermal Gravimetric Analysis (TGA)

Thermal gravimetric analysis was used to measure the thermal stability of the obtained chitooligomers fractions and the unmodified chitosan. A TGA 5500 thermogravimetric analyser from TA Instruments Company (Tokyo, Japan) was used. The analyses were carried out by increasing the temperature from room temperature to 300 °C in an inert nitrogen atmosphere with a flow rate of 25 mL/min and a warming rate of 10 K/min.

### 2.8. Nuclear Magnetic Resonance (NMR)

Nuclear magnetic resonance measurements of solid state with cross-polarisation spinning under magic-angle technique CP MAS NMR were performed on an Avance III 400 MHz spectrometer (Bruker Corporation, Billerica, MA, USA). The spectra for the ^13^C nuclei were taken with 100.61 MHz frequency and for the ^1^H nuclei with 400.15 MHz frequency in the MAS BB DVT, which enabled zirconia rotors (ZrO_2_) with a 4 mm diameter. Isotopically labelled L-tyrosine (Tyr) was used to select the parameters for ^13^C CP MAS NMR spectra, including optimising for Hartmanna–Hahna conditions. The most important spectroscopic parameters were as follows: ^13^C CP MAS spectra, measurement temperature 298 K, rotation speed 8 kHz, relaxation time 3 s, pulse 90″ for ^1^H 4 µs, contact time 2 ms, spectral width SWH = 40 kHz, TD = 3.5 k, SPINAL decoupling type; ^1^H MAS spectra: measurement temperature 298 K, rotation speed 8 kHz, relaxation time 2 s, pulse 90″ for ^1^H 4 µs, spectral width SWH = 40 kHz, TD = 16 k. The baseline intensity was corrected by subtracting the average noise intensity of the lactic acid used to dissolve chitosan (–C=O—180.12 ppm; –CH_3_—20.47 ppm).

### 2.9. Matrix-Assisted Laser Desorption/Ionisation Technique with Time of Flight Analyser (MALDI-TOF)

The samples were prepared by mixing 10 µL of the test sample with 10 µL of matrix solution (aqueous solution of 2.5-dihydroxybenzoic acid at a concentration of 10 mg/mL). Subsequently, 1 µL of the prepared solution was applied on a plate to accelerate solvent evaporation. In the next step, the plate was introduced into the Axima-Performance TOF mass spectrometer ion source (Shimadzu Biotech, Manchester, UK). As the ionisation technique, a desorption/ionization matrix-assisted laser (matrix-assisted laser desorption/ionization—MALDI) was used. The wavelength of the laser radiation—337 nm, the energy of the laser beam—was slightly above the level needed to obtain the signals; the accelerating voltage (20 kV) was set. Positive ions were subjected to registration using the time of the flight of ions analyser (time of flight—TOF); the recorded mass range (*m*/*z*) was between 300 and 3500. The mass spectrum was the sum of 200 spectra, each consisting of two laser shots from randomly selected sampling points (200 spectra × 2 shots = 400 laser shots). External mass calibration was used, performed on the basis of a defined reference mixture of the polyethylene glycol spectrum.

### 2.10. DPPH Radical Scavenging Activity

The free radical scavenging activity of the hydrolysates was determined by the method of Chen et al. [17]. A hydrolysate of 1% was 20-times diluted in water, and 1.0 mL was taken and mixed with 1.0 mL of 0.1 mM 2,2-diphenyl-1-picrylhydrazyl (DPPH)–methanol solution. The reaction mixture was shaken well and incubated for 30 min at room temperature. The reduction in DPPH free radical was measured by reading the absorbance at 517 nm. The control sample was a DPPH–methanol solution. Antioxidant activity was calculated from the standard curve and expressed in µM Trolox/mL needed to neutralize a 0.15 mM solution of DPPH free radicals.

### 2.11. Antimicrobial Assay

The antimicrobial effect was tested using the broth microdilution method against *Escherichia coli* (PCM 2560). Microorganism strains were obtained from the culture collections from the Institute of Immunology and Experimental Therapy (Polish Academy of Sciences, Wroclaw, Poland). The experiment was carried out according to Alqahtani et al. [18] with modifications. The microorganisms were grown in Mueller-Hinton Broth (MHB) (Merck, Poznan, Poland) for 18 h at 37 °C and adjusted to 1 on the McFarland standard. Then, 10 µL of the bacterial broth suspension was inoculated into each well containing 10 µL of NF, HMF, LMF, and CH in 230 µL of MHB and again incubated for 18 h at 37 °C. Bacterial growth was measured every 0.5 h using a microplate reader (Bioscreen C, Growth Curves USA, Piscataway, NJ, USA) set at an optical density (OD) of 600 nm for 48 h. 

### 2.12. Statistical Analysis

A completely randomised block design was performed to analyse the data with the analysis of variance (ANOVA) using Statistica 13 (TIBCO Software Inc., Palto Alto, CA, USA). Differences between means were identified using Duncan’s test with a confidence level at *p* < 0.05. Two-way factor analysis of variance was used to analyse the data (variables: type of enzyme and type of fraction). All statistical analyses were made after the normality (Shapiro–Wilk test) and homogeneity (Levene test) of variances was confirmed.

## 3. Results and Discussion

### 3.1. Enzymatic Reaction Progress

**Figure 1:**
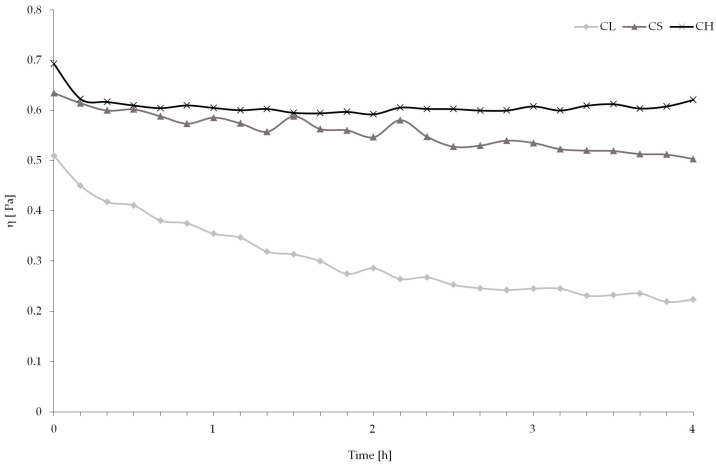
Dynamic viscosity during CL and CS hydrolysis and the CH sample (not treated with enzyme) during 4 h of reaction.

The greatest reduction of viscosity during enzymatic hydrolysis was observed in cellulase-treated samples. After 3.5 h of hydrolysis, flattening of the viscosity curve was observed, suggesting that the whole dose of enzyme reacted with the substrate (Figure 1). Xie [19] also confirmed a rapid reduction in viscosity in chitosan degraded by a cellulase/pectin/α-amylase (1:1:1) complex enzyme. Chitosanase was not that effective at the assumed time of the experiment. Fen et al. [20] proved that rapid drop in viscosity was observed until the eighth hour of hydrolysis of chitosan by chitosanase when the viscosity remained relatively constant until the end of the experiment. Nguen et al. [21] observed that degradation of chitosan by D4 chitosanase from *P. janthinellum* continued until almost 50 h of hydrolysis.

The hydrolysis process increased the reducing sugar content in enzyme-treated samples (average: 0.40 mM glucosamine for cellulase and chitosanase treatment) compared to untreated chitosan (0.28 mM glucosamine), which together with dynamic viscosity reduction, confirmed the appearance of the enzymatic hydrolysis process.

### 3.2. Apparent Viscosity

**Figure 2:**
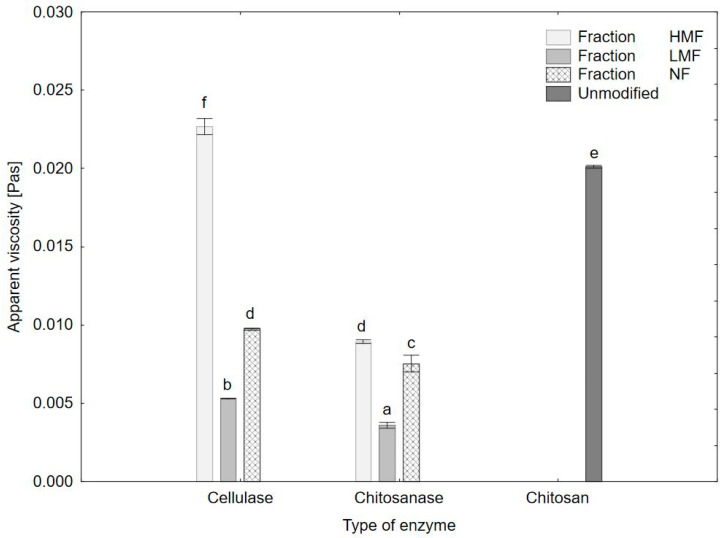
Average apparent viscosity of COS and unmodified chitosan sample. The results are expressed as the mean ± standard error. Values with different letters (a–f) within the same column differ significantly (*p* < 0.05).

The apparent viscosity of the fractions obtained was measured after hydration of the freeze-dried samples (Figure 2). The LMF fraction was characterized by the lowest dynamic viscosity, whereas HMF was characterised by the highest values of Pa. In comparison to cellulase fraction HMF, LMF, and NF and unmodified chitosan, fractions made with the use of chitosanase were characterised by much lower viscosity values, suggesting different reaction mechanism of that enzyme preparation.

### 3.3. Water Solubility and pH Measurements

The insoluble nature of chitosan at neutral pH restricts its use in the medical and food industries. Therefore, in the present work, it was demonstrated that the applied conditions of enzymatic hydrolysis of chitosan made it possible to obtain preparations of chitooligomers with very good solubility in water, without the need to use an acidic medium (Table 1). The use of chitosanase and cellulase to the same extent increased the preparation of solubility of the chitooligomers (average solubility: 83.47%) compared to chitosan dissolved in pure water (11.85%). The HMF fractions were characterised by a lower degree of solubility in water (61.57%), while the LMF and NF fractions were characterized by improved solubility in water at a level of 94.56% in comparison to the unmodified chitosan sample (89.31%) dissolved using 1% lactic acid. No effect of the type of enzymes used on the solubility in water of the obtained COS was demonstrated. The lowest pH values were obtained for the CL_LMF sample (3.86) and the unmodified chitosan sample dissolved in lactic acid (3.75) (Table 1). In addition to poor water solubility, HMF fractions were characterised by elevated pH values (6.01). The improved solubility of the LMF and NF samples may have been the result of the simultaneous action of two factors: the lower molecular weight of the obtained chitooligomers, confirmed by MALDI-TOF analysis, and chitosan protonation by lactic acid. Protonation of the amino groups of chitosan can lead to the formation of COS lactate salt, as indicated by the low pH values of these samples. Li et al. [22] indicated that the pH value has a great influence on the antimicrobial properties of COS. The protonation of free amino groups in COS may play an important role in the binding process to the bacterial membrane and thus enhanced the antibacterial activity. In this paper, the authors’ hypothesis was confirmed, as LMF samples with lower pH values (and better solubility in water) exhibited improved anti-microbiological properties in comparison to HMF samples. 

### 3.4. Thermo-Gravimetric Analysis (TGA)

**Figure 3:**
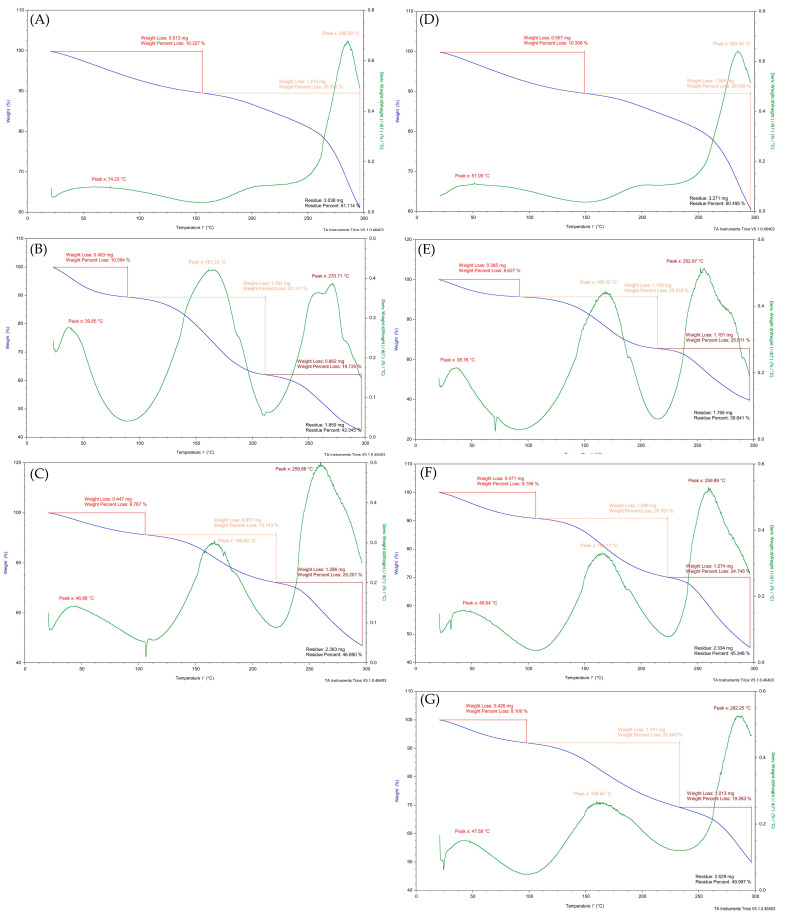
TGA decomposition curves of (**A**) CL_HMF, (**B**) CL_LMF, (**C**) CL_NF, (**D**) CS_HMF, (**E**) CS_LMF, (**F**) CS_NF, and (**G**) CH.

The thermal decomposition process of the CL and CS fractions (HMF, LMF, NF) and unmodified chitosan was evaluated by thermogravimetric analysis (TGA). The decomposition curves (Figure 3) have been summarised in Table 2, taking into account the highest values for the temperature curves (inflection points) and the mass losses related to these peaks during thermal treatment. The results obtained show that HMF made with the use of CL and CS enzymes underwent a two-stage thermal degradation process, while chitosan, as well as LMF and NF samples, were degraded in a three-stage process (Table 2). There were no significant differences between samples hydrolysed with the use of CL and CS enzymes. Differences in the peak area and placement in TGA curves indicate that the obtained materials differed in their WHC (water holding capacity) and strength of water to polymer interaction. The water molecules can be bound by hydroxyl and amine polar groups, both presented in the chitosan structure. Rueda et al. [23] and Neto et al. [24] confirmed that the interaction of water with the hydroxyl groups is stronger than with the amine groups. Because a significant amount of water is released at temperatures below 100 °C, it can be concluded that the water molecules should be mainly bound to the amine groups and, consequently, simply removed at lower temperatures than those molecules bound to the hydroxyl groups [24]. According to above, the first stage of the decomposition process occurred between 39.94 °C and 74.06 °C, which was due to the loss of mass through vaporisation of the absorbed moisture and weakly bound water through amine groups [24,25]. It was observed that HMF samples needed a higher temperature to release absorbed water than LMF and NF fractions, which indicates the role of macromolecular chains in the process of water absorption by these polymers and the strength of water–polymer interaction. The second stage of thermal degradation occurred at 159–168 °C and showed the release of water bound to the polar hydroxyl groups [24,26]. No peak in the HMF samples in the region of 159–168 °C indicates that most of the water in these samples was bounded by amine groups not hydroxyl groups, and on the other hand, LMF, NF, and CH samples had a better ability to retain water and, as a result, these samples had a better predisposition to create hydrogel structures. The third stage of thermal degradation, visible as an inflection point near 252–284 °C, was connected to depolymerisation of chitosan and chitooligomers and degradation of their pyranose rings. The thermal stability of individual fractions was determined by their mass losses during temperature treatment. The greatest mass loss was recorded for LMF samples ($\bar{x}$ = 58.35%) which suggests their sensitivity to high-temperatures treatments. HMF was the most resistant to decomposition, and the NF and CH samples were characterised by intermediate thermal stability properties.

### 3.5. Nuclear Magnetic Resonance (NMR)

**Figure 4:**
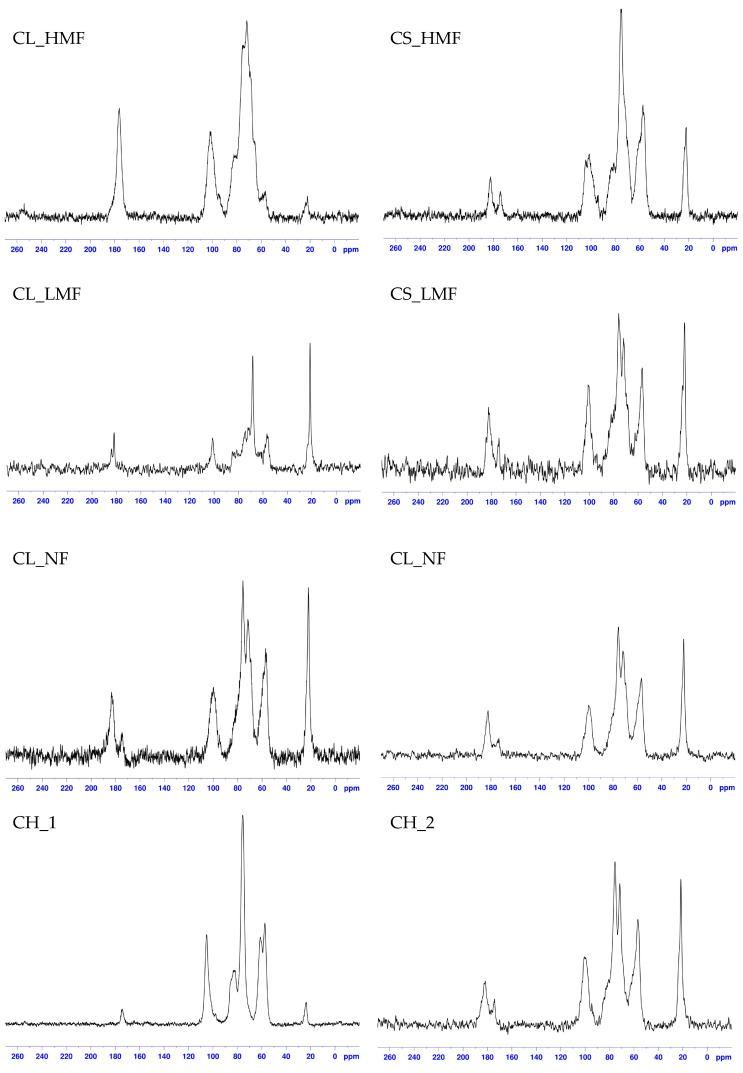
CPMAS ^13^C NMR spectra of chitosan hydrolysis products (different fractions of hydrolysis with cellulase: CL_LMF, CL_HMF, CL_NF and chitosanase; CS_LMF, CS_HMF, CS_NF) and unmodified chitosan CH_1 and its lyophilised acid solution, CH_2.

CPMAS ^13^C NMR spectra of chitosan hydrolysis products and unmodified chitosan are presented in Figure 4, and their chemical shift values (δ) are shown in Table 3. All spectra showed peaks assigned to carbonyl carbon –COCH_3_ of the acetyl group GlcNac from the structure of the chitosan between 174 and 181 ppm. A peak centred at 100 ppm is characteristic of the C1 carbon assigned to the acetylated amino group from the chitosan structure. Peak intensities in the region 56–81 ppm were assigned to C2–C6 chitosan glucopyranose carbons. No methanol residue was observed in the analysed samples (should be visible as a peak centred at 50 ppm). A slight shift in spectra signals was observed compared to native chitosan sample, which may confirm changes in the chemical structure of chitooligomeric products (Figure 4). An increase in the peak intensities corresponding to CH_3_ (22.0 ppm) and –C=O (near ≈ 174–181 ppm) of chitooligomers compared to those signals of native chitosan confirmed increased values of the degree of acetylation (DA) values of these products. An increase in these peaks was also observed in the studies conducted by Kumar et al. [27] where chito-oligomeric-monomeric mixtures were obtained using papain and Pronase enzymes. They suggested that those enzymes cleaved only the glycosidic bonds, leaving the N-acetyl group intact, thus confirming their depolymerising action rather than a deacetylating effect. Chitosanase activity (EC 3.2.1.132) is the endohydrolysis of β-(1,4) linkages between N-acetyl-D-glucosamine (GlcNac) and D-glucosamine (GlcN) residues in partially deacetylated chitosan, which was confirmed by an increasing degree of acetylation in hydrolysis products in this study. On the other hand, cellulase, which belongs to GH-8, is limited to cleave GlcN-GlcN bonds exclusively, which is the reason why DA remained at a level quite similar to that of unmodified chitosan. Li et al. [28] suggested that the DD of the initial chitosan can also affect the enzymatic hydrolysis and thus the characteristic of the COS, which may make it difficult to discuss the results between individual authors.

### 3.6. Matrix-Assisted Laser Desorption/Ionization Time-Of-Flight (MALDI-TOF)

**Figure 5:**
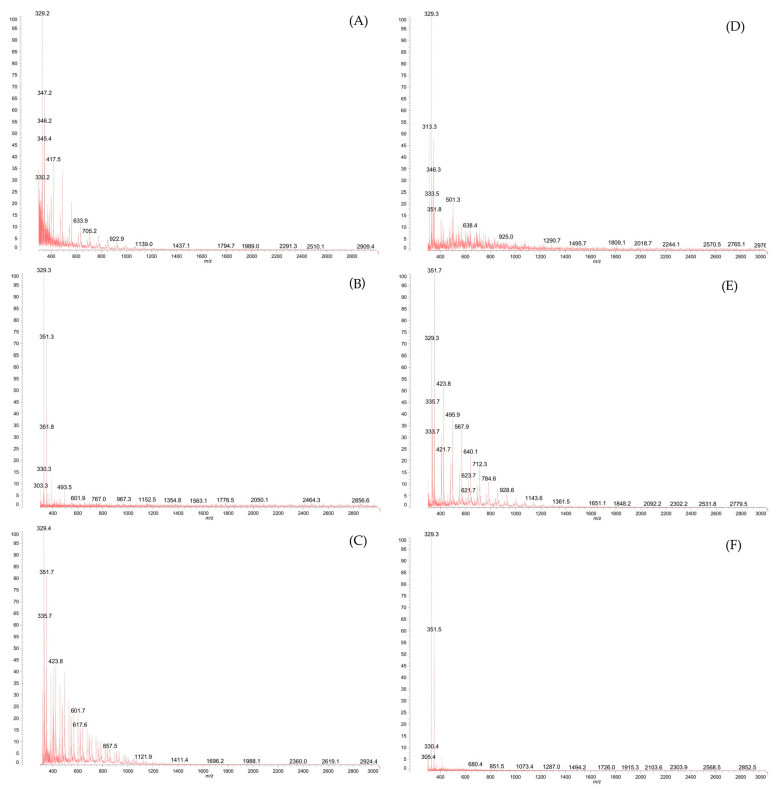
MALDI-TOF spectra of (**A**) CL_HMF, (**B**) CL_LMF, (**C**) CL_NF and (**D**) CS_HMF, (**E**) CS_LMF, and (**F**) CS_NF.

Matrix-assisted laser desorption/ionisation time-of-flight (MALDI-TOF) analysis is a popular technique for profiling chitooligosaccharides. MALDI-TOF spectra (Figure 5A–D) showed a series of characteristic ion peaks of multimers with different DPs formed by the combination of GlcN (D-glucosamine) and GlcNAc (N-acetyl-D-glucosamine). After the hydrolysis reaction, the average molecular weight of all analysed samples was <3 kDa, which confirmed the successful degradation of chitosan (initial Mw = 50–190 kDa). The cellulase and chitosanase reaction products were a mixture of heterochitooligomers with DPs of 3–18. Based on the peaks concentration in the 300–600 *m*/*z* region of the CL_HMF and CL_NF spectra (Figure 5A,C) in comparison to CS_HMF and CS_NF (Figure 5D,F) it can be already seen that cellulase fractions consisted of more low-DP products than COS made with the use of chitosanase. Furthermore, the relative ion intensity can reflect the quantification of the products [29]. The greatest intensities of signals in CL_HMF, CL_NF, and CS_HMF, CL_NF were observed in range of 500–1200 *m*/*z*, which suggests concentrations of products with DPs of 3–7. The low-molecular-weight fractions (LMF) obtained by the reaction with CL and CS showed a reduced number of peaks concentrated in the 300–400 *m*/*z* region, which may suggest that this fraction consisted of products with DP~2. Unfortunately, as described in the work of Liang et al. [29], molecular weights less than 500 Da cannot be analysed due to the possibility of matrix signal interference; consequently, chitooligomers with DP < 2 could not be determined by this method. Chen et al. [30] used MALDI-TOF MS to analyse the distribution of N-acetyl-D-glucosamine and deacetylated glucosamine units in COS with degrees of polymerisation (DPs) in the range of 5–12.

### 3.7. DPPH Radical Scavenging Activity

**Figure 6:**
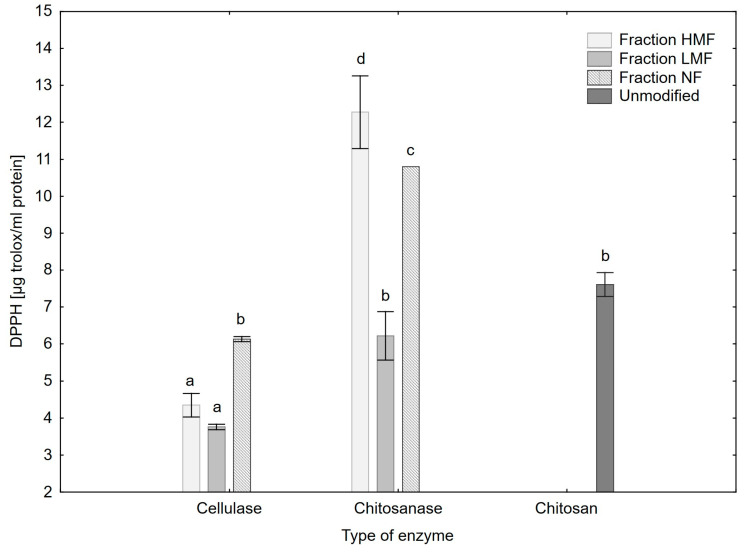
DPPH radical scavenging activity of the hydrolysis products and the chitosan unmodified sample. The results are expressed as the mean ± standard error. Values with different letters (a–d) within the same column differ significantly (*p* < 0.05).

The mechanisms of the antioxidant action of chitosan and chitooligosaccharides were previously described by Xue et al. [31]. The authors pointed out that the scavenging abilities of chitosan derivatives against the ^•^OH radical are the result of: (1) the reaction of the free amino groups with the radical; (2) the formation of NH_3_^+^ through addiction reaction of the radical with NH_2_; or (3) the hydrogen atom abstraction reaction between the hydroxyl groups in the polysaccharide unit and the hydroxyl radical [31]. The hydroxyl and superoxide radical scavenging activity of chitosan and COS have been reported to increase with a decreasing value of the average molecular weight (Mw) [32,33,34,35,36,37]. However, it was found that the superoxide radical scavenging activity of COS characterised with Mw < 3000 Da and low DP is not subject to the rule mentioned above. Yang et al. [37] reported that COS with Mw = 1100 Da had higher superoxide radical scavenging activity than COS with 500 Da. Similarly, Park et al. [33] demonstrated that COS (average Mw = 2000 Da) showed stronger superoxide radical scavenging activity than COS with Mw = 1000 Da. Li et al. [38] confirmed that COS with high DP exhibited a stronger ability to scavenge superoxide radical than that with low DP. Consistent with the above results, it was observed that COS fractions (Mw < 3000 Da) characterised by DP~2 (LMF) showed lower DPPH radical scavenging activity than HMF and NF chitooligomers, with narrower DP concentrated between 3 and 7 (Figure 6). In addition to the above, cellulase-treated samples (CL_HMF, CL_LMF, CL_NF) exhibited lower antioxidant potential than fractions made with the use of chitosanase. MALDI-TOF analysis confirmed that cellulase treated samples were characterised by more low-DP products than COS made with the use of chitosanase, which also confirms the above theory. The inferior antioxidant potential of cellulase hydrolysed samples may also be associated with a lower concentration of N-acetyl-D-glucosamine units and what follows: a lower concentration of hydrogen ions from the NH_3_^+^ groups, which can react with free radicals. The more NH_3_^+^ groups, the better the prevention of the oxidation process of lipid-rich food products, especially those containing unsaturated fatty acids (USFA); therefore, CS_HMF and CS_LMF samples have application potential in industrial food production.

### 3.8. Antimicrobial Assay

**Figure 7:**
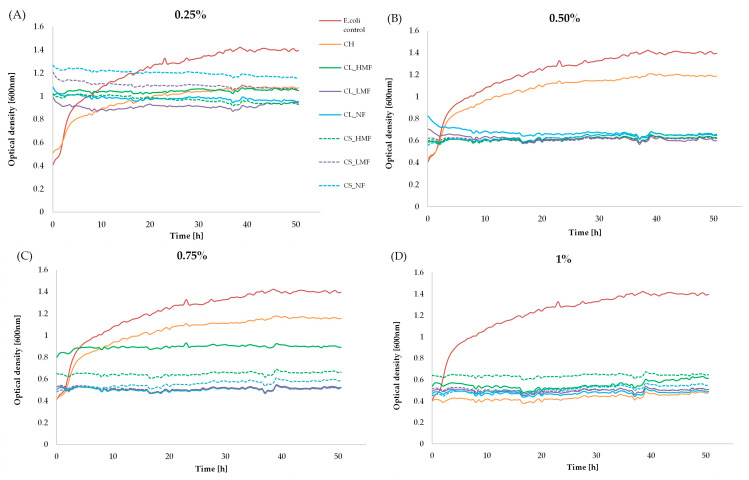
*Escherichia coli* growth curves in the presence and absence of chitosan hydrolysis products (different fractions of hydrolysis with cellulase: CL_LMF, CL_HMF, CL_NF and chitosanase; CS_LMF, CS_HMF, CS_NF) and unmodified chitosan CH at the concentration of (**A**) 0.25%, (**B**) 0.5%, (**C**) 0.75%, and (**D**) 1%.

Guan and Feng [39] presented several mechanisms underlying the antimicrobial activity of chitosan and COS: (1) suppression of bacteria by combining with the components with negative charges on the bacterial surface (e.g., peptidoglycans in the cell walls of Gram-positive bacteria or O-antigens of lipopolysaccharide in the outer membranes of Gram-negative bacteria) through their protonated amino group with positive charges at acidic pH; (2) chelating by the free amino groups the metal ions on the cell surface of bacteria to form complexes, thus preventing the growth of the bacteria by cutting off the supply of minerals; (3) the ability of low-molecular-weight COS and chitosan with low DP to enter the bacterial cell and penetrate the nucleoid. Figure 7 shows the antimicrobial activities of chitosan (CH) and different fractions of its hydrolysis products by cellulase: CL_LMF, CL_HMF, CL_NF and chitosanase; CS_LMF, CS_HMF, and CS_NF against *Escherichia coli* strains. The smaller the optical density (OD) of the medium was, the higher the antibacterial activity of the tested material was. As can be seen, the analysed population of the 0.25, 0.50, 0.75, and 1.0% (*w*/*v*) chitosan-treated microorganisms was less than that of the control throughout the 50 h of incubation period, which confirms its antimicrobial potential. On the other hand, it was confirmed that the products of enzymatic hydrolysis of chitosan by cellulase and chitosanase exhibited a higher inhibitory growth activity against *E. coli* than untreated chitosan sample. The ability to reduce *Escherichia coli* increased in the given order: LMF > NF > HMF > CH. The LMF and NF fractions obtained in CL hydrolysis showed a higher antimicrobial potential in comparison to the same fractions obtained with the help of the CS enzyme. In contrast, CS_HMF possessed a higher ability to reduce *E. coli* strain than CL_HMF. The results obtained suggest that unlike in the antioxidant properties observations, COS with lower DP are characterised by better antimicrobial properties than COS consisting mostly by higher-DP products. This was confirmed by the fact that the LMF fraction possessed the greatest antimicrobial properties (DP~2), and COS obtained by the CL enzyme generally exhibited greater *E. coli* reduction features than the CS samples. 

## 4. Conclusions

The enzymatic hydrolysis of chitosan by cellulase and chitosanase is a promising method that may be used to achieve functional and biologically active chitosan oligomers. The use of various enzymes resulted in a different course of the enzymatic reaction, which was confirmed by observations of changes in viscosity during the analysis, the concentration of reducing sugars, and the CPMAS ^13^C NMR spectra of produced fractions. The use of commercial and dedicated enzymes affects the products of the enzymatic reaction and their properties. The fractionation of the enzymatic reaction products allowed us to confirm that the fractions differed by DP (2–7 DP range), which, in turn, affected their biological activity. LMFs were characterised by stronger antimicrobial properties than HMF fractions, which, on the other hand, were characterised by better free radical scavenging abilities than LMF. LMF fractions were characterised by enhanced solubility in water medium but unfortunately demonstrated deteriorated thermal stability compared to HMF, which may limit their use in processes where the temperature is higher than 200 °C. To reduce the cost of the process, it is possible to omit the fractionation stage and thus obtain a chitosan hydrolysis product (NF) characterised by intermediate but still improved biological activities, enhanced solubility in aqueous solutions (an average 94.73% of the solubility rate compared to unmodified chitosan, which has a solubility in water of 11.85%), and optimum thermal stability (8% lower mass loss during TGA thermal treatment than LMF samples). Upgraded biological and functional features enable industrial applications of such hydrolysates, such as the pharmaceutical or food industry for, e.g., extending the shelf life of food products sensitive to oxidation and the development of microorganisms like products of animal origin.

## Figures and Tables

**Table 1:** Solubility and pH values of HMF, LMF, and NF made with the use of CL and CS enzymes and unmodified chitosan (CH).

Sample	Solubility (%)	pH
CL_HMF	61.04 ^a^ ± 0.21	6.06 ^d^ ± 0.19
CL_LMF	93.88 ^c^ ± 1.22	3.86 ^a^ ± 0.13
CL_NF	95.38 ^c^ ± 1.07	4.88 ^c^ ± 0.11
CS_HMF	61.57 ^a^ ± 1.15	5.97 ^d^ ± 0.11
CS_LMF	94.92 ^c^ ± 0.08	4.29 ^b^ ± 0.02
CS_NF	94.08 ^c^ ± 0.18	4.87 ^c^ ± 0.05
CH (1)	89.31 ^b^ ± 1.34	3.75 ^a^ ± 0.17

^a–d^ values with different letters within the same column differ significantly (*p* < 0.05). ^(1)^ chitosan dissolved in 1% lactic acid solution, solubility of chitosan in water—11.85%.

**Table 2:** Thermal decomposition temperatures, mass loss, and residues of HMF, LMF, and NF made with the use of the CL and CS enzyme and unmodified chitosan (CH).

Sample	Inflection Point 1 (°C)	Inflection Point 2 (°C)	Inflection Point 3 (°C)	Mass Loss 1 (%)	Mass Loss 2 (%)	Mass Loss 3 (%)	Residue (%)
CL_HMF	74.06 ^f^ ± 0.24	N	284.88 ^e^ ± 0.30	10.06 ^c^ ± 0.37	N	28.14 ^d^ ± 0.33	61.03 ^f^ ± 0.11
CL_LMF	39.94 ^b^ ± 0.13	161.11 ^b^ ± 0.30	270.30 ^c^ ± 0.57	10.35 ^c^ ± 0.33	27.10 ^e^ ± 0.29	19.40 ^a^ ± 0.46	41.93 ^b^ ± 0.57
CL_NF	47.23 ^c^ ± 0.49	166.53 ^c^ ± 0.42	259.55 ^b^ ± 0.46	8.64 ^b^ ± 0.18	19.06 ^a^ ± 0.11	25.03 ^bc^ ± 0.24	46.60 ^d^ ± 0.41
CS_HMF	50.93 ^e^ ± 0.22	N	284.52 ^e^ ± 0.04	10.16 ^c^ ± 0.21	N	28.85 ^d^ ± 0.22	60.12 ^f^ ± 0.52
CS_LMF	38.84 ^a^ ± 0.11	168.03 ^d^ ± 0.40	252.77 ^a^ ± 0.28	8.52 ^ab^ ± 0.15	25.74 ^d^ ± 0.25	25.57 ^c^ ± 0.34	39.37 ^a^ ± 0.37
CS_NF	48.72 ^d^ ± 0.11	165.87 ^c^ ± 0.33	260.11 ^b^ ± 0.32	9.09 ^b^ ± 0.10	20.54 ^b^ ± 0.29	24.38 ^b^ ± 0.51	44.97 ^c^ ± 0.53
CH	47.50 ^c^ ± 0.11	159.49 ^a^ ± 0.49	282.47 ^d^ ± 0.32	8.02 ^a^ ± 0.11	22.45 ^c^ ± 0.26	19.00 ^a^ ± 0.36	49.33 ^e^ ± 0.47

N—no peak observed, ^a–f^ values with different letters within the same column differ significantly (*p* < 0.05).

**Table 3:** CPMAS ^13^C NMR chemical shift values (***δ***) of chitosan hydrolysis products (different fractions of hydrolysis with cellulase: CL_LMF, CL_HMF, CL_NF, and chitosanase; CS_LMF, CS_HMF, CS_NF) and CH_1 and its lyophilised acid solution, CH_2.

Sample	*δ* (ppm)							
	–C=O	C1	C4	C5	C3	C6	C2	–CH_3_
CL_HMF	176.36	101.94	81.00	75.25	71.73	58.99	57.42	22.10
CL_LMF	181.91	101.36	76.77	75.71	68.43	61.63	56.83	21.43
CL_NF	183.29	99.74	75.69	74.65	69.04	59.32	57.77	22.15
CS_HMF	182.45	102.51	76.01	74.96	69.16	59.54	57.53	22.35
CS_LMF	182.47	100.85	75.75	74.78	69.64	58.04	56.53	21.87
CS_NF	182.34	101.25	76.48	75.55	71.89	57.57	56.60	22.07
CH_1	174.21	105.00	81.78	76.53	74.95	61.21	57.37	23.54
CH_2	176.36	101.94	81.00	75.25	71.73	58.99	57.42	22.10

## Data Availability

The data presented in this study are available on request from the corresponding author.
